# Claudins in lung diseases

**DOI:** 10.1186/1465-9921-12-70

**Published:** 2011-05-27

**Authors:** Ylermi Soini

**Affiliations:** 1 Department of Pathology and Forensic Medicine, Institute of Clinical Medicine, Pathology and Forensic Medicine, School of Medicine, University of Eastern Finland, Cancer Center of Eastern Finland, P.O. Box 1627, FI-70211 Kuopio, Finland

## Abstract

Tight junctions are the most apically localized part of the epithelial junctional complex. They regulate the permeability and polarity of cell layers and create compartments in cell membranes. Claudins are structural molecules of tight junctions. There are 27 claudins known, and expression of different claudins is responsible for changes in the electrolyte and solute permeability in cells layers. Studies have shown that claudins and tight junctions also protect multicellular organisms from infections and that some infectious agents may use claudins as targets to invade and weaken the host's defense. In neoplastic diseases, claudin expression may be up- or downregulated. Since their expression is associated with specific tumor types or with specific locations of tumors to a certain degree, they can, in a restricted sense, also be used as tumor markers. However, the regulation of claudin expression is complex involving growth factors and integrins, protein kinases, proto-oncogens and transcription factors. In this review, the significance of claudins is discussed in lung disease and development.

## Tight junctions

**Figure 1:**
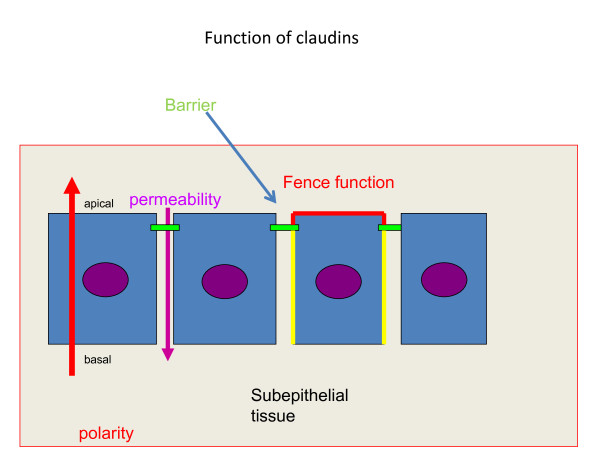
**In an epithelial cell layer tight junctions (marked by green) regulate the permeability of solutes and ions through the paracellular space (a violet arrow pointing downwards)**. Thus they function as barriers of the paracellular space and also prevent pathogens from reaching the subepithelial tissues. Tight junction also takes part in determining the polarity of epithelial cells (red arrow). Since they are located in the apicolateral part of the cell membrane they separate the apical part of the cell membrane (red) from the lateral part (yellow) preventing membrane proteins from these parts of mixing up with each other (the fence function).

Tight junctions are membranous structures present in epithelial, endothelial and mesothelial cells [1,2] They form barriers between cells in cell layers regulating diffusion of molecules and ions through the intercellular space [3]. They also form a fence separating the apical part of the cell from other parts thus preventing membrane proteins from mixing up with each other [3]. They play a part in the formation of cellular polarity and attachment [2,3] (Figure 1). The barrier formed by the tight junctions prevents pathogens from penetrating through the epithelial layers thus serving as components of innate immunity [4]. They also participate in the immune defense by forming secluded areas such as the brain, eye or testis, where they contribute to sealing these tissues from the immune system [5,6]. Tight junctional proteins also participate in regulation of cell differentiation and proliferation [7].

**Figure 2:**
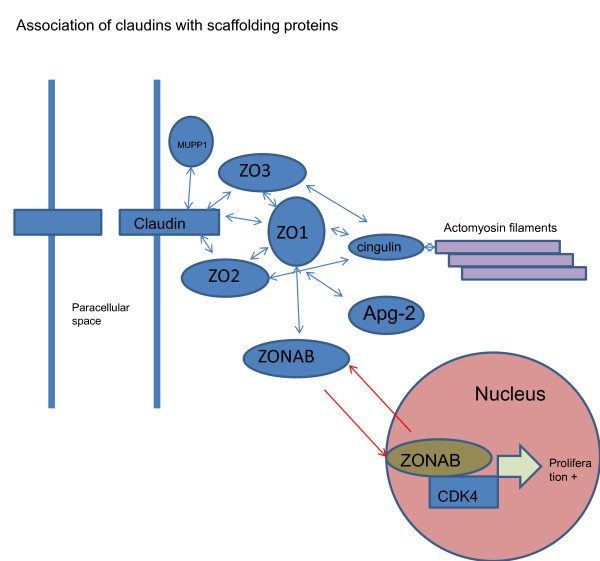
**Interaction of claudins with scaffolding proteins ZO-1, ZO-2, ZO-3, MUPP1, cingulin and ZONAB in tight junctional structures**. The arrows show interactions between specific molecules. If ZONAB is released from the association with ZO-1 it moves to the nucleus and binds to cyclin dependent kinase 4 (CDK 4) resulting in cellular proliferation. Apg-2 competes with ZONAB for the same binding site in ZO-1 thus affecting the binding of ZONAB to ZO-1 and releasing it to the nucleus. This is one example how tight junction proteins may influence the functions of the cell

Tight junctions consist on one hand of membrane proteins mediating cell to cell contacts. They include occludin, claudins, tricellulin and JAMs (junctional adhesion molecules). Scaffolding proteins on the other hand mediate signals from the surface to cytoskeletal actin filaments and activate signaling cascades of the cell [1,3]. They include ZO-1 (Zona occludens-1), ZO-2, ZO-3, MAGI-1 (membrane-associated guanylate kinase with inverted orientation-1), cingulin and MUPP1 (multi-PDZ domain protein 1) [1,3,7]. Membrane proteins are divided in two groups, those with one transmembrane domain (JAMs) and those with four (claudins, occludin, tricellulin) [7]. The membrane proteins contain sequences in their carboxyterminal end with which they can attach to the scaffolding proteins [7]. One such is the PDZ domain with which the membrane proteins bind to ZO-1, ZO-2, ZO-3 or MUPP1 proteins [7,8]. Scaffolding proteins, like ZO-1 inhibit cell proliferation by binding ZONAB (ZO-1 associated nucleic acid binding protein) thus preventing its movement to the nucleus [7]. Apg-2 (Albino and pale green 2), a protein involved in heat shock reaction, may replace ZONAB from its association with ZO-1 thus promoting its movement to the nucleus resulting in increased proliferation [7,9] (Figure 2). Also ZO-2 influences cell proliferation by binding to transcription factors AP-1 (Activator protein 1) and SAF-B (Scaffolding attachment factor B) [7].

## Claudins

**Figure 3:**
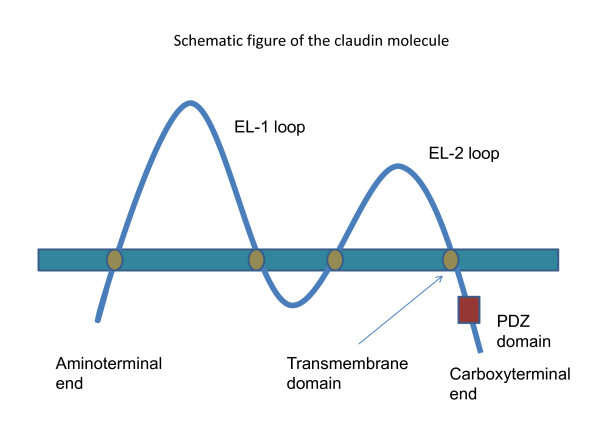
**Claudins are membranous proteins harbouring two extracellular loops (EL-1 and EL-2) and one small intracellular loop**. The larger EL-1 loop regulates solute permeability and charge selectivity while the smaller EL-2 loop establishes contacts with the neigbouring cell. There are four transmembrane domains in claudin molecules. By the carboxyterminal part the claudin molecule attaches to the scaffolding proteins of the tight junctions.

Claudins are proteins responsible for the regulation of the paracellular permeability of cells [1,3]. They were discovered in 1998 by Tsukida and Furuse [10] and currently 27 claudins are known to be expressed by mammals[1,11]. In humans, claudin 13 is missing [12]. Based on their sequence similarity they are divided into classic and non-classic claudins [1,12]. The former include claudins 1-10, 14, 15, 17 and 19 and the latter claudins 11-13, 16, 18 and 20-24 [1]. Claudins have four transmembrane domains, between these there are two extracellular loops (EL1 and EL2) and between inner transmembrane domains there is a short 20-residue intracellular loop [1,12] (Figure 3). The intracellular carboxyterminal part contains the PDZ domains by which scaffolding proteins attach to claudins [1,12]. The larger EL1 loop influences paracellular charge selectivity and the smaller EL2 binds the claudin molecule to the corresponding one in the neighbouring cell [12]. Claudins can associate with the same claudin or another one on the same cell membrane or with the claudin of the neighbouring cell [1,13]. Heterodimerization between claudins can take place only between specific claudins, for instance between claudin 1 and 4 but not between claudin 1 and 2 [13]. In general, claudins 2, 7, 10, 15 and 16 increase paracellular cation permeability by forming pores in the tight junctions whereas claudins 4, 5, 8, 11, 14 and 18 have a sealing function [1].

## Claudins in normal lung

Claudins 1, 3, 4, 5, 7, 8 and 18 are expressed in human bronchi and bronchioles but there are discrepant reports of the expression of claudin 2 [14-18]. In immunohistochemical studies on formalin fixed human lung tissue bronchial epithelial cells expressed claudins 2 [17,18]. Similarly, claudin 2 has been detected in mouse lung [15]. However, in mRNA analysis of bronchial tissues it was not found [14]. Claudin 2 has been detected in skin squamous cells and in enterocytes where it takes part in absorption of D-vitamin from the intestine [19,20]. High amounts of claudin 2 mRNA are found in kidney, pancreas, stomach and liver tissues while neural and lymphatic tissues lacked expression [21]. In the study of Aung et al trace amounts of claudin 2 were found in the lung but the source of the expression was not determined [21].

Claudin expression appears to be the same in larger and smaller bronchi and there is no variation in claudin expression as has been found in different parts of the gut or kidney tubular segments [14,22,23]. Quantitative differences may, however be present and claudins appear to be located not only in the apical region of cells but also on the lateral membrane [14]. Claudin 5 is considered to be expressed mainly in endothelial cells and it takes part in the formation of the blood brain barrier [24]. It appears to be involved in the sealing function of endothelial barriers in others sites such as blood retinal or blood testis barrier [5,6]. When claudin 5 was overexpressed in bronchial tranfectant cells it, however, made the epithelium leakier while claudins 1 and 3 made it tighter [14]. In rat alveolar cells, EGF stimulation increased the transmembrane resistance with an increased claudin 4 or 7 protein expression while claudins 3 and 5 were associated with a leakier phenotype [25]. Paradoxically then, claudin 5 appears to induce loosening of the barrier both in bronchial and alveolar cells [14,25].

In immunohistochemical studies on human alveolar cells, claudins 3, 4 and 7 have been detected in type 2 alveolar cells while type 1 alveolar cells were negative [17,18]. Rat alveolar epithelial cells expressed claudin 3, 4 and 7 mRNA but claudin 1 or claudin 5 mRNA were not found even though claudin 5 was found in western blot analysis in alveolar cells [25]. Rat type 2 alveolar cells express more claudin 3 and type 1 cells more claudin 7 [16,26]. Thus there are inconsistencies regarding claudin expression also in alveolar cells detected by immunohistochemistry or mRNA analysis which may be species specific, partly due to the method used or may reflect extended half lives of proteins compared to RNA. Inconsistent results between protein and mRNA levels of claudins 1, 3 and 7 have also been detected in alcohol induced lung changes in rats where the protein expression of these claudins increased while the mRNA levels stayed the same [27]. In studies on claudin mRNA expression in human lung tissues claudins 6, 9, 10, 11, 15 or 16 have not been detected [14,16]. Expression of claudins in lung cells has been compiled in Table 1.

**Table 1:** Claudin expression in bronchial, alveolar and mesothelial cells

	Claudin protein or mRNA expression	Reference
Bronchial cells	**1**, 2, **3**, **4**, **5**, **7**, 8*, 18*	[14-18,81,82]
Alveolar cells type 2	2, **3, 4,5,7**, 18 1*	[15-18,25-27,81]
Alveolar cells type 1	1,3,4,5,7,8,18	[27,68,26]
Mesothelial cells	1,2, 3,5,7	[104,107]

* = only mRNA expression found

bold = both mRNA and protein expression found

normal text = only protein expression found

## Claudins in embryonic development of the lung

Claudins are involved in embryogenesis and organogenesis and changes in their expression can be seen in the organogenesis of the gut and kidney [23,28]. Claudin 15 serves as a target for tcf2 gene in zebrafish creating one lumen instead of multiple ones [29]. Interestingly, claudin 15 knockout mice develop a megaintestine [30]. In organogenesis, claudin expression is turned off in cells taking part in epitheliomesenchymal transition [31]. In embryonic development claudins regulate cellular integrity, and create hydrostatic pressure differentials maintaining integrity of cystic compartments [31,32]. Additionally, claudin 1 may have a role in right-left patterning of the embryonic tissues [31]. In Xenopus, claudin 5 is required for heart development [33].

There are five stages in lung organogenesis. In the embryonic period, the tracheal bud forms. The pseudoglandular and canalicular periods are characterized by branching morphogenesis giving rise to the bronchial three while during the saccular and alveolar periods the alveoli form[17]. In investigations on claudin expression during these stages claudins 1,3,4, 5 and 7 can be detected in bronchial epithelium during the pseudoglandular and canalicular periods [17]. Pretype alveolar cells express claudins 3, 4, 5 and 7 but not claudin 1[17]. In saccular and alveolar periods claudin 5 is lost from alveolar cells but weakly present in bronchial epithelial cells. Claudins 3, 4 and 7 show strong positivity in bronchial and alveolar cells while claudin 1 is negative in alveolar pneumocytes [17].

Compared to lung development also kidney ureters develops by first forming an ureteral bud followed by branching morphogenesis. Like in the lung tracheal budding ZO-1 is required in ureter bud formation of rat tissues [28]. Branching morphogenesis is promoted by ezrin which is activated by growth factors EGF (Epidermal growth factor), HGF (Hepatic growth factor) or interleukin-1alpha by tyrosine phosphorylation. MT-MMP1 (Membrane type matrix metalloproteinase 1) and MMP2 (Matrix metalloproteinase 2) are secreted on tips of the epithelial branches and proliferation is higher in epithelial cells in those areas [28]. Proteins of adherence junctions such as E-cadherin, are expressed a bit earlier, however there is a gradual increase of claudin 3 and ZO-1 from day 3 to 5 in cultured ureteric buds [28]. Anatomically, the branching morphogenesis in the lung differs from the ureteric development in that in the ureteral development there is no lateral branching [28]. Also EGF (Epidermal growth factor) seems to be important for lung organogenesis, deletion of EGFR (Epidermal growth factor receptor) leads to impaired branching and deficient alveolisation, and pneumocytes remain immature [34].

Prealveolar fetal alveolar lung cells express claudin-1, 3, 4, 5, 7, 9, 10 and 18 mRNAs [16]. mRNAs for occludin, JAM-1, ZO-1, ZO-2, and ZO-3 are also present [16]. In experimental models on alveolar cell differentiation undifferentiated fetal alveolar epithelial cells increase their transepithelial resistance under the influence of cell culture medium containing dexamethasone, 8-bromo-cAMP and isobuthylmethylxanthanine and transform to type 2 alveolar cells at the same time increasing the claudin 5 and 18 mRNA levels and decreasing claudin 1 mRNA [16]. On the other hand, EGF stimulated fetal alveolar type 2 cells show a decrease in claudin 5 and 3 mRNA but an increase in claudins 4 and 7 mRNA while increasing their transepithelial resistance and transforming to type 1 cells [25]. While such in vitro models may differ from physiological conditions, changes in claudin expression apparently modulate or follow phenotypic changes in lung bronchial or alveolar cells during organogenesis. Such studies also emphasize EGF's role in lung alveolar development.

## Potential roles of claudins and tight junctions in lung disease

Tight junctions are important for lung defense since they function as a barrier for pathogens and other exogenous compounds preventing their penetration into the interstitial tissues. Tight junctions may thus be considered as a part of the innate immune system. The epithelial barriers of bronchi are, however, relatively leaky with a comparatively low transmembrane resistance of approximately 100 Ωcm2 compared to endothelial cell junctions of the blood brain barrier which have a 1500-2000 Ωcm2 barrier resistance [14,35]. The thickness of the tight junctional belt in airway epithelia varies from 0.27 μm to 0.37 μm depending on the cell type [36]. There are reports of fragmentation and thinning of the tight junctional belt in diseases such as asthma and focal proliferations and thinnings are described in lung neoplasia such as bronchioloalveolar carcinoma [36]. Such changes are also seen in cell line studies induced by cytokines [37]

Lungs are exposed to several pathogens and noxious stimuli which may influence paracellular permeability of bronchial airways, alveolar cells or endothelial barriers in alveolar walls. Tobacco smoke makes tight junctions leakier [36]. Carcinogenic compounds such as bentzpyrene and 4-(methylnitrosamino)-1-(3-pyridyl)-1-butanone (NNK) influence claudin synthesis by upregulating transcription factors like twist, snail or ZEB1 [38,39] resulting in downregulation of E-cadherin and claudins. Exposure of lung BEAS-2B cells and cancer cell lines to tobacco smoke leads to changes in claudin expression [40]. In bronchial cells sampled from smoking COPD patients and healthy smokers claudins and other tight junction genes are downregulated [41]. Tobacco smoke also induces the formation of reactive oxygen species. In kidney epithelial MDCK (Madine-Darby canine kidney strain) II cells, exposure to hydrogen peroxide leads to bimodal changes in the transepithelial barrier permeability with activation of ERK1/2 and p38 kinases, and a decrease in occludin, claudin 1 and claudin 2 levels [42]. Such changes could also make the bronchial and alveolar epithelium leakier and the airways more vulnerable to damage caused by pathogens causing irritation and chronic infection. On the other hand, an increase in claudin 2 expression leads to lower transepithelial resistance in MDCK I and II cells and formation of paracellular water channels [43,44]. Generally air pollutants influence the barrier function of airway epithelium. Particulate matter of less than 10 μm in diameter decreases the transepithelial potential in airway epithelial cells causing internalization of occludin and its dissociation from ZO-1 [45].

Inflammation may affect the barrier function of tight junctions. Several integrins and growth factors produced by inflammatory cells influence synthesis or organization of tight junction components leading to enhanced permeability and exposure of tissues to antigens [15,46,47]. Increased permeability of tight junctions is one factor lying behind the pathophysiology of colitis ulcerosa and Crohn's disease [47]. Also in chronic bronchitis and asthma airway epithelial cells are exposed to chronic stimulation by integrins and growth factors predisposing the bronchial wall to dysregulation of the epithelial barrier function further provoking the chronic infection [46,48-50].

Pathogens may influence tight junctions in two ways. Several bacteria and viruses lower the transepithelial resistance by decreasing the expression of tight junctional proteins making it easier for pathogens to penetrate the tissues. Rhinovirus downregulates the transepithelial resistance of nasal epithelial cells by lowering the mRNA expression of claudin 1, ZO-1, E-Cadherin and occludin [51]. HIV-1 decreases the transepithelial resistance in enterocytes and genital epithelial cells by disrupting claudins 1, 2, 4, occludin and ZO-1 in tight junctions and lowering mRNA expression of claudins 1, 2, 3, 4, 5, occludin and ZO-1 by cytokine involved mechanisms allowing penetration of bacteria and viruses through the leaky barriers [52]. Rotavirus in known to loosen tight junctions in gut epithelial cells by decreasing expression of claudin 3, occluding and ZO-1 [53]. *Yersinia *enterocolica causes a redistribution of claudins 3, 4 and 8 in colonic HT29/B6 cells with a lowering of transepithelial resistance and diminution of the protein expression of claudins 2, 3, 8 and 10 and ZO-1 [54]. Aggressive strains of *E *Coli cause dissociation of claudin 1, ZO-1 and occludin from cell membranes along with a decrease in electrical transmembrane potential [55]. *Helicobacter *pylori causes disruption of claudin 4 expression in tight junctions and its movement to the cytosol in gastric epithelial cells and lowering of claudin 4 protein levels, an effect which is dependent on IL-1β [56]. On the other hand, claudins may be used as receptors or co-receptors in the invasion of bacteria and viruses to cells and tissues. Claudins 1, 6 and 9 serve as a co-receptors for hepatitis C for invading liver and endothelial cells [57,58]. Claudin 1 also promotes dengue virus entry to cells [59]. Claudins also serve as receptors for some bacterial toxins. A well known example is *Clostridium *perfringens enterotoxin (CPE) which binds to claudin 3 and 4 [60]. In experimental studies this has been used to destroy claudin 3 and 4 expressing cancer cells with chemical modifications of the toxin [60]. Interestingly, CPE has also been successfully used to destroy metastatic cells expressing claudin 4 in murine tissue [61]. Unfortunately, claudin 4 is many times downregulated in metastatic tissues such as metastatic breast carcinoma [62] or carcinoma metastasis to the lung [63]. The frequent associations of claudins as means and targets for spread of infections could predict putative polymorphisms in claudin molecules. Indeed, 50 variations have been found in the claudin 1 gene region some of which alter susceptibility to hepatitis C infection [64].

Mechanisms of pathogen entry and its association with claudins have not been so extensively studied in the respiratory tract. It, however, appears probable that similar mechanisms apply to airway epithelia. Matrix metalloproteinase 9 (MMP 9)decreases epithelial barrier function in bronchial cells, and increases their vulnerability to adenovirus infection [65]. The cells show disruption of regular membranous staining pattern of claudin 1 and occludin and internalization of reactivity following MMP9 treatment which could be restored by tissue inhibitor of matrix metalloproteinase 1 (TIMP1) [65]. *Pseudomonas *aeruginosa has been shown to invade airway epithelial barriers by destroying tight junctions [66]. Interestingly, *Plasmodium *falciparum malaria affects lung endothelial cells by relocation of ZO-1 from the plasma membrane and downregulation of claudin 5 [67]. Thus also parasite infections may be associated with tight junctional changes in the pulmonary cells.

## Claudins in lung injury and inflammation

ARDS (Acute respiratory distress syndrome) is due to injury of alveolar cells the reasons of which may be multiple including septic infections and toxic chemicals. Such injury leads to edema due to leakage of alveolar epithelial cells. Since claudins regulate paracellular permeability of cell layers evidently changes in claudin expression are involved in such disease. There are no morphological studies on claudin expression by human tissues in ARDS. Evidence of tight junctional protein changes in ARDS can be obtained from animal experiments or cell culture studies. In alveolar cells cultured from septic rat lung a decrease of claudin 4, claudin 18 and occludin was found associated with a decrease in transmembrane resistance [68]. In ventilator-induced lung injury in mice the mRNA expression of claudin 4 increased after 3 hours [69]. Such a phenomenon appears to be an adaptive mechanism to make the alveolar layers tighter, since blocking specifically the mRNA synthesis of claudin 4 by siRNA or CPE lowers the transmembrane potential [69]. Similar results come from experimentally induced pancreatitis in rats where the mRNA levels of claudin 4, claudin 5 and occludin decreased due to pancreatitis induced lung injury but increased after administration of emodin, a chemical suggested to enhance epithelial barrier function [70]. Such data emphasize especially the role of claudin 4 in combating the abrogation of alveolar cell permeability in lung injury. Also the expression of claudin 5 is important for endothelial cell tightness and vascular permeability. Acrolein present in tobacco smoke may cause lung injury by influencing claudin 5 expression in endothelial cells and mice more resistant to acrolein have a better survival due to their increased expression of claudin 5 mRNA transcripts [71].

Interestingly, protein kinase C delta inhibitor appeared to inhibit sepsis induced lung injury, such as disruption of lung tissue or development of edema [72]. In nasal epithelial cells treatment of cells with protein kinase C activator 12-O-tetradecanoylophospho-13-acetate led to an increased transepithelial electrical resistance and upregulation of claudin 1, ZO1, ZO2 and occludin [73]. It was shown that the upregulation of the tight junctional proteins was due to activation of PKC lambda and theta partly induced by the transcription factor GATA 3 [73]. In fact, phosphorylation of threonine sites in claudins by PKC or PKA may dislocate claudins from cellular membranes resulting in changed tight junctional properties [1].

Also in lung inflammation alterations in alveolar cell permeability play a role. In experimental studies on mice where lung inflammation was induced by carrageenan disruption of claudin 2, claudin 4 and claudin 5 staining and ZO-1 was observed on cell membranes [15]. Such disruption was partly reversed by blocking TNFα suggesting that TNFα-induced chemotaxis of inflammatory cells might contribute to attenuation of tight junctional permeability in lung inflammation [15]. Additionally, alcohol which provokes patients to lung inflammation and ARDS was shown in experiments on rats to induce changes in claudin expression of alveolar epithelial cells [27]. Chronic alcohol ingestion of rats led to a decrease of claudin 1, 3 and 7 mRNA and protein expression and an increase in claudin 5 mRNA in alveolar epithelial cells resulting in an increased leakiness of tight junctions in the lung [27]. Additionally, inflammatory mediators like IL-1β or IL-6 decrease claudin 3 and 4 levels in amniotic membranes of mice making them more permeable [74].

Respiratory distress leads to local hypoxia of the lung. Hypoxia induces cytoskeletal disruption of alveolar epithelial cells and decreases ZO-1 protein levels dislocating occludin from the cell membrane [75]. Alveolar epithelial cells do not show changes in claudin 3 or 5 protein expression, however [75]. Claudin 5 is known to influence the permeability of endothelial cells. In experiments performed on mice bEND.3 (brain-derived endothelial) and retinal endothelial cells, suppression of claudin 5 mRNA leads to a decrease in transepithelial resistance of these cells [76]. On the other hand, in experimental murine renal ischemia-reperfusion injury there was a significant upregulation of claudin -1, -3, and -7 genes and a slight downregulation of claudin-2 [77]. Additionally, when both of these cells suffer from hypoxia a decrease in claudin 5 mRNA and a lowering of transepithelial resistance was detected [76].

There are only a few studies on the influence of inflammation on claudin expression in bronchial cells. In well differentiated human airway epithelial cells (HAE) from cystic fibrosis (CF) and non-CF patients, combined treatment with IL-1β and TNFα led to a decrease in transmembrane potential and tight junctional barrier function, the changes appearing more rapidly in CF cells [37]. In analysis of airway epithelial cells the expression of claudins 1 and 4 were not altered, however, there was a decrease in the protein expression of ZO1 and JAM and upregulation of hyperphosphorylated occludin and ICAM1 [37]. The influence of these cytokines appeared to be mediated by PKC/lambda [37].

## Claudins in interstitial lung disease

Interstitial lung diseases are characterized by chronic inflammation, fibrosis and damage to the lung parenchyma [78]. They consist of seven entities which are classified both by histology and clinicopathologic features [78]. In interstitial lung diseases, reports of claudin expression in UIP (Usual interstitial pneumonia) are present [18]. Changes in claudin expression mainly involve the regenerative alveolar cells or cells which have been replaced by metaplastic epithelia. In UIP regenerative metaplastic alveolar, squamous or bronchial epithelium shows strong reactivity for claudins 1, 2, 3, 4 and 7 compared to normal alveolar cells where only claudins 3, 4 and 7 are present [18]. There is a weaker reactivity for claudin 5 in such regenerative cells. In sarcoidosis, metaplastic regenerative epithelium displays a similar kind of reactivity in metaplastic cells as in UIP [18]. The formation of metaplastic epithelium in UIP probably is related to chronic inflammation and secretion of growth factors and integrins in the disease [78]. A different pattern of claudin expression in regenerative metaplastic cells compared to alveolar cells would lead to focal changes in the permeability of alveolar walls which is probably harmful for the function of the lung. There is also increased vascularity, neovascularisation and increased secretion of angiogenic factors in UIP [18]. Endothelial cells of blood vessels display intensive expression for claudin 5. According to some reports VEGF may upregulate claudin 5 [79]. Such increased staining may then be based on increased secretion of VEGF in UIP. Expression of claudins in different lung pathologies has been compiled in Table 2.

**Table 2:** Claudin expression changes in different lung pathologies

	UIP*	ARDS	Inflammation	Lung SQC**	Lung AC**	Lung SCC**	Reference
CL1	↑		↔	↑↔	↔↔	↔	[37,18,81,82,47,76,77]
Cl2	↑		↓	↔↔	↔↔	↔	[15,18,81,82]
CL3	↑		↓	↔↔	↔↔	↑	[18,81,82,82]
CL4	↑	↓	↓↔	↔↔	↑↑	↑	[15,18,37,68-70,81,82]
CL5	↑	↓	↓	↓	↓		[15,68-70,81,82]
CL7	↑			↔↔	↑↔	↔	[18,81,82,82]

CL = Claudin; UIP = Usual interstitial pneumonia; ARDS = acute respiratory distress syndrome; SQC = Squamous cell carcinoma; AC = Adenocarcinoma; SCC = Small cell carcinoma

* = arrows represent metaplastic alveolar cells

** = qtRNA results compared with lung tissue expression

## Lung tumors

Lung carcinoma cells contain tight junctions and their number is inversely associated with patient prognosis and aggressivity [80]. In lung tumor cell lines, variable expression of claudins 1, 2, 3, 4 and 7 is found [40]. In studies on lung tumor material different histological tumor types vary in their claudin expression showing up- or downregulation of different claudins compared to normal bronchial cells or lung tissue [40,81-83]. In the study of Moldway et al small cell lung carcinomas show a 16 fold higher level of claudin 3 mRNA expression than normal lung tissue [82]. Claudin 4 mRNA is upregulated to 3-4 fold level in squamous, adenocarcinoma and small cell carcinoma compared to normal lung [82]. Both adeno- and squamous cell carcinoma showed a slight downregulation of claudin 1 mRNA compared to normal lung and squamous cell carcinomas had a 2.7 fold level of claudin 1 mRNA than adenocarcinomas [82]. Paschoud et al found an increase in claudin 5 and a decrease in claudins 3, 4 and 7 mRNA in squamous cell carcinoma compared to normal bronchial cells and an increase in claudin 1 and a decrease in claudin 5 compared to lung parenchyma [81]. Compared to bronchial cells claudins 1, 3 and 4 and 7 were decreased and claudin 5 mRNA increased in adenocarcinoma while when compared to lung parenchyma claudin 4 mRNA was increased but claudin 5 decreased [81].

In immunohistochemical studies significant differences have been detected in claudin 3 expression between squamous cell and adenocarcinomas the latter displaying stronger expression [40,82,83]. Some studies also report significant differences in the expression of claudin 1, 4, 5 and 7 between these two tumor groups [81,82]. In the study of Paschoud et al squamous cells and adenocarcinomas could be discriminated by the expression of claudins 1 and 5 because adenocarcinomas displayed strong positivity for claudin 5 and weak positivity for claudin 1, while the opposite could be detected in squamous cell carcinomas [81]. Chao et al studied claudin 1 expression in adenocarcinoma and found that a low expression of claudin 1 was associated with a worse survival in these tumors both by immunohistochemistry and mRNA expression [84]. Transfection of claudin 1 into CL1-5 lung carcinoma cells changed the cells to less invasive, less metastatic and less mobile, and their morphology changed to a more epithelial one [84]. Such changes could also be reversed by blocking claudin 1 mRNA expression by siRNA. Oligonucleotide microarray analysis showed a twofold change in 773 genes due to claudin 1 overexpression involving different cellular functions associated with signaling cascades, cellular associations, apoptosis and cytoskeletal regulation. They, however, found that claudin 1 overexpressing cells were able to activate MMP2 [84]. Similar results of claudin expression on MMP activities have been detected in other cell lines. In colon carcinoma CaCo-2 cells, invasion induced by claudin 4 overexpression was due to activation of both MMP2 and MMP9 the mRNA expression of which also increased at the same time [85]. Claudin 5 along with claudins 2, 3 and 4 has been shown to activate matrix metalloproteinases [86]. Claudin overexpression in cancer cells may thus be one factor promoting tumor spread.

The variable expression of claudins in histologically different lung tumor types may be related partly to the cell type it originates from [83]. On the other hand, several growth factors and integrins modify claudin expression and they expression may vary in different types of lung tumors. EGF, for instance, inhibits claudin 2 expression while simultaneously increasing claudin 1, 3 and 4 expression and the transepithelial resistance without affecting the levels of occludin or ZO-1 in canine kidney cells [87]. In pneumocytes EGF upregulates claudins 4 and 7 while 3 and 5 are decreased [25]. Activating EGFR mutations are known to exist in non-small cell lung carcinoma and especially in adenocarcinoma and bronchioloalveolar carcinoma[88]. In line with this, claudins 1 and 4 show a high expression in lung adenocarcinomas and bronchioloalveolar carcinomas and claudin 2 and 5 are lower [40,82]. Small cell lung carcinomas also display a higher expression of claudin 2 than adenocarcinomas [83]. Another mutation common to lung cancer is K-Ras [88]. Such mutations are present 15-30% of non small cell carcinomas [88]. In kidney cells, overexpression of ras leads to claudin 1, 4 and 7 overexpression while claudin 2 decreases and claudins 3 and 5 remain the same [89]. Similarly, some non-small cell carcinomas have PTEN (phosphatase and tensin homologue) mutations [90]. Inactivation or mutations of PTEN leads to activation of PKB (Protein kinase B) resulting in increased cell proliferation and inhibition of apoptosis [91]. Knockdown of PTEN has been shown to lead to a loss of polarization in colon carcinoma cells and strong downregulation of claudins 1, 3, 4 and 8 [92]. Small cell carcinomas and large cell neuroendocrine carcinomas, however, express c-kit, and they harbor c-myc amplifications and p53 and ras mutations, and loss of p16 and RB expression and their influence on claudin expression is not known [93]. Additional, several cytokines influence claudin expression and barrier function of epithelial cells and may surely influence also the expression of claudins in lung carcinomas [47].

EMT (Epitheliomesenchymal transition) is a process where tumor cells attain mesenchymal features which make it easier for them to invade and metastasize [94,95]. It is regulated by transcription factors such as snail, slug, twist and zeb1 [94-96]. In mouse mammary epithelial cells snail induced EMT and at the same time downregulated claudins 3, 4 and 7, occludin and E-cadherin [97]. In lung carcinoma there was an inverse association between zeb1 and claudins 1 and 2 and between twist and claudin 5 [63]. Metastatic tumors to the lung also showed an inverse association between claudins 5 and 7 and twist [63]. These results show that a part of claudin expression is regulated by EMT associated transcription factors and that claudins are involved in EMT.

Also different PKCs which regulate claudin phosphorylation are overexpressed in cancer and may cause dysregulation or relocation of claudins in tumor cells [98]. PKCε is considered an oncogene and influences cellular motility and ras expression, and it is upregulated in lung and breast cancer [98]. PKC and PKA influence subcellular distribution of claudin 1 in melanoma cells [99]. PKCα phosphorylates claudin 5 causing its disappearance from cell membrane [100]. On the other hand, phosphorylation of serine 194 of claudin 4 by aPKC is required for tight junction formation in keratinocytes [101]. Claudin phosphorylation may also affect barrier junction permeability without changes in claudin distribution [101].

Claudins are in contact with scaffolding proteins like ZO-1, ZO-2 and ZO-3 and through them with the cellular cytoskeleton [102]. ZO-1 has been shown to recruit the transcriptional factor ZONAB from cytoplasm thus preventing its movement to the nucleus where it promotes cell proliferation. ZO-2, on the other hand, is able to bind AP-1 [103]. How the expression of different claudins influences the functions of such zona occludens proteins is not known but it is possible that in cancer cells the relation between claudins and zona occludens and other scaffolding proteins is deranged leading to a dysregulation of transcription factors such as ZONAB or AP-1. Clearly more research is needed to understand the functions and consequences of claudin expression in lung cancer.

Because claudins 3 and 4 are overexpressed in adeno, squamous and small cell carcinomas [40,81-83] they might be susceptible to CPE-mediated treatment. In a recent publication Yao et al created a modified CPE protein containing segments of pseudomonas aeruginosa enterotoxin A [104]. Treatment of cell lines containing claudin 4 induced apoptosis and cell destruction in them[105]. CPE also causes dislocation of claudin 4 from the tight junction in ovarian carcinoma cells and sensitizes them to chemotherapeutic drugs [105]. This effect is achieved by using a modulated C- terminal fragment of the CPE molecule in the treatment which is able to bind to claudin 4 and cause tight junctional loosening but harbours no toxic effects of the N-terminal part of the molecule thus enabling a better influx of chemotherapeutic agents to the cancer tissue (105). Such CPE mediated treatment could also be one option in treatment of claudin 3 or 4 positive lung cancer.

## Mesothelial cells and mesotheliomas

Mesotheliomas are malignant tumors with an aggressive behavior. In pleural biopsies taken for malignant pleural disease, there is often diagnostic difficulty to distinguish mesotheliomas from metastatic adenocarcinomas or reactive mesothelial cells. Several markers, such as calretinin, DJ2-40, TTF1 and CEA, can be used in differential diagnosis [106]. Claudins have also proven to be one putative means of making a differential diagnosis between such entities [107-110].

In our study on metastatic adenocarcinomas and malignant mesotheliomas claudins 1, 3, 4, 5 and 7 had a lower expression in mesotheliomas suggesting that they could serve as differential diagnostic markers [107]. In mesothelioma subtypes, sarcomatoid and biphasic mesotheliomas showed less expression for claudins than epithelioid ones [107]. Non-neoplastic mesothelial cells showed expression for claudin 2 and weak expression for claudin 1, but no expression was found for claudins 3, 4, 5 or 7 [107]. In effusions claudins 3 and 7 significantly distinguished malignant

Mesothelioma cells from metastatic adenocarcinoma cells [108]. Reactive mesothelial cells were negative for claudin 7 but showed infrequent claudin 1 and 3 expression. Thus also reactive mesothelial cells could be distinguished from metastatic cells in pleural effusions [108]. Metastatic ovarian carcinoma cells could also be distinguished from metastatic breast carcinoma cells by claudin 7, and generally, ovarian, endometrioid and cervical cancer metastases could be distinguished from lung or breast adenocarcinoma [108]. In the array study by Davidson et al serous mesotheliomas showed a significantly lower expression of claudins 3, 4 and 6 than ovarian adenocarcinomas speaking for a lower level of claudins in mesothelial derived neoplasms [108]. Facetti et al determined that especially claudin 4 could be used in differential diagnosis between malignant mesothelioma and metastatic adenocarcinoma [110]. Since adenocarcinoma metastases many times express claudin 4, CPE could potentially be used in treatment of such adenocarcinoma metastases in pleural cavity since benign mesothelial cells do not express this tight junctional protein [110].

Claudins have not been studied in other disease states of the mesothelium although it could be expected that they play a role in derangements of pleural fluid homeostasis and in pleuritis. Exposure of mesothelial cell monolayers to hydrogen peroxide lowers their transepithelial resistance with a simultaneous decrease in occludin and ZO-1 expression [111]. Thus oxidative stress, involved in many diseases, such as inflammation, may be one factor causing loosening of mesothelial barriers. In line with this, a recent article showed that the levels claudins 1, 3, 5 and 7 decreased and claudin 2 increased in mesothelial cells due to pleural inflammation [112].

## Conclusion

The permeability of tight junctions in epithelia is an important factor in several pulmonary diseases. Pathogens causing infections are many some of which may act by downregulating claudins or causing their deranged distribution inducing changes in tight junctional permeability allowing pathogens to invade through epithelial barriers. In lung disease the effect of such pathogens on claudin expression or cellular distribution is still mainly unelucidated and needs further research. Alteration of claudin expression also plays an important role in lung diseases such as COPD, asthma,and ARDS. Influencing alveolar or endothelial permeability by manipulation the expression of claudins (eg. claudins 4 and 5) might be future targets in the treatment of lung injury. Changes in the expression of claudins are also seen in pulmonary neoplasia reflecting complex changes in several genes related to tumor growth, spread and differentiation producing a characteristic expression patterns in histologically different tumor types. In adenocarcinomas a proposed new classification will surely also change our perspectives on assessment of claudins on the behavior of these tumors [113]. In addition to claudin 3 and 4 which may have therapeutic implications for tumor treatment in the future, claudin 18 might also be a new target for antibody mediated therapy in lung cancer [114].

## Competing interests

The author declares that they have no competing interests.

## Authors' contributions

YS is the sole author of the manuscript
